# Expression of Human Endogenous Retrovirus-K in Spinal and Bulbar Muscular Atrophy

**DOI:** 10.3389/fneur.2019.00968

**Published:** 2019-09-04

**Authors:** Cody Rex, Marie-Josée Nadeau, Renée Douville, Kerri Schellenberg

**Affiliations:** ^1^Department of Biology, University of Winnipeg, Winnipeg, MB, Canada; ^2^Department of Immunology, University of Manitoba, Winnipeg, MB, Canada; ^3^Division of Neurology, University of Saskatchewan, Saskatoon, SK, Canada

**Keywords:** endogenous retrovirus-K (ERVK), spinal and bulbar muscular atrophy (SBMA), amyotrophic lateral sclerosis (ALS), peripheral blood mononuclear cells (PBMC), inflammation, DNA damage, antivirals

## Abstract

**Background:** Spinal and Bulbar Muscular Atrophy (SBMA) is caused by the extension of the polyglutamine tract within the androgen receptor (AR) gene, and results in a multisystem presentation, including the degeneration of lower motor neurons. The androgen receptor (AR) is known to modulate the expression of endogenous retrovirus-K (ERVK), a pathogenic viral genomic symbiont. Since ERVK is associated with motor neuron disease, such as Amyotrophic Lateral Sclerosis (ALS), we sought to determine if patients with SBMA exhibit evidence of ERVK reactivation.

**Results:** Data from a pilot study demonstrate that peripheral blood mononuclear cell (PBMC) samples from controls and patients with SBMA were examined *ex vivo* for the expression of ERVK viral transcripts and proteins. No differences in ERVK RNA expression was observed between the clinical groups. In contrast, enhancement of processed ERVK Gag and integrase proteins were observed in SBMA-derived PBMC as compared to healthy control specimens. Increased ERVK protein maturation co-occurred with elevation in the expression of the pro-inflammatory transcription factor IRF1 in SBMA.

**Conclusions:** Our findings indicate that ERVK viral protein maturation in SBMA is an unrecognized biomarker and facet of the disease. We discuss how our current understanding of ERVK-driven pathology may tie into key aspects of multi-system dysfunction in SBMA, with a focus on inflammation, proteinopathy, as well as DNA damage and repair.

## Background

Spinal and bulbar muscular atrophy (SBMA, also called Kennedy's disease) is a rare motor neuron disease with X-linked recessive inheritance. This adult onset neuromuscular disorder clinically presents with progressive lower motor neuron dysfunction, skeletal muscle wasting, and accompanying multi-organ involvement (1, 2). SBMA pathology is driven by a polyglutamine (polyCAG) tract expansion in the androgen receptor (AR) gene, resulting in polyglutamine (polyQ) repeats in the protein product. Notably, several other neurological conditions with cognitive and motor involvement are associated with polyQ expansions: *huntingtin* in Huntington's disease (3), *ATAXIN-2* in spinocerebellar ataxia 2 (SCA2) (4) and its pre-mutation expansion with Amyotrophic lateral sclerosis (ALS) (5, 6), *Atrophin-1* in Dentatorubral pallidoluysian atrophy (DRPLA) and several genes implicated in distinct types of spinocerebellar ataxia (4). Generational trinucleotide repeat expansions in risk genes can lead to offspring with earlier disease onset and more severe clinical symptoms in SBMA (2, 7). PolyQ expansions in excess of 37 amino acids long are considered pathogenic (8).

Trinucleotide repeat expansion disorders (TREDs) such as SBMA have been associated with both loss-of-function and gain-of-function effects (9). It remains uncertain how the CAG repeat expansion induces neurodegeneration in SBMA. However, similar to all other polyglutamine diseases, an accumulation of intranuclear inclusions with misfolded polyglutamine-expanded proteins are found in certain neuronal populations. In SBMA cases, these deposits can occur in the anterior horn motor neurons of the spinal cord (10). The pathogenic mechanism is not well-understood, but increasing evidence suggests that toxicity of the mutant AR protein is due primarily to androgen-dependent impairment of its receptor, as well as having impacts in terms of proteinopathy. When androgen binds mutant AR, this leads to AR aggregation, nuclear inclusions in certain tissues, and altered function as a transcriptional regulator (11–13).

Mutant polyQ proteins can accumulate into proteinaceous deposits in neural cells, as well as peripheral cell types, such as muscle (14) and immune cells (15). Our work has previously demonstrated that aggregate-prone, mutated TDP-43 protein can facilitate the accumulation of endogenous viral proteins within cells (16). Amazingly, over 8% of human DNA is of retroviral origin—scattered inside our genome are thousands of retrovirus-like sequences called endogenous retroviruses (ERVs) (17, 18). When activated by signals such as inflammation, select pre-existing viruses in our DNA can produce viral proteins within human cells (19). The cellular consequences related to the expression of these viral proteins is largely unknown. However, accumulating evidence points toward endogenous retrovirus-K (ERVK) driving neurodegeneration in ALS (19–22). Therefore, due to overlapping cellular mechanisms and aspects of clinical presentation, we postulated that ERVK expression may be enhanced in SBMA, as seen in ALS.

## Methods

### Ethics Statement

All research involving human-derived samples was approved by the University of Winnipeg Human Research Ethics Board under a multi-site protocol GT916 (R1) and the University of Saskatchewan Biomedical Research Ethics Board under protocol 17-26. All participants consented to blood donation, and samples were anonymized by clinician Dr. Kerri Schellenberg prior to processing by the Douville lab.

### Diagnosis and Demographics of Participant Samples

Clinical examination and genetic screening for CAG repeats in AR was used to confirm the clinical diagnosis of SBMA. Genetic testing was not performed on control specimens. Table 1 indicates the individual patient diagnosis and number of CAG repeats in AR for the samples used in this study.

**Table 1:** PolyQ expansions in SMBA patients.

**Clinical group**	**Identifier**	**Sex**	**Age**	**Number of CAG repeats in *AR***
Controls	03	MM	36	Unknown
	05	M	56	
	07	M	44	
	09	M	43	
SBMA	01	M	49	60
	02	M	50	58
	06	M	60	52
	08	M	33	55

### PBMC Isolation

Whole blood samples were diluted in saline solution and processed on a ficoll gradient (GE Healthcare 17-5442-02) as previously described (23). The time from collection to processing for all patient samples was <20 h. Extracted *ex vivo* PBMC were counted and aliquoted into 5 x 10^6^ cells per dry pellet and frozen until subsequent batched analysis.

### Quantitative Polymerase Chain Reaction (Q-PCR)

Total RNA was extracted and purified from cells using an Aurum Total RNA Mini Kit (Bio-Rad #732-6820) and Q-PCR performed as previously described using SYBR Green detection method (19). The primers used were: ERVK gag F: 5′-TCGGGAAACGAGCAAAGG-3′ and R: 5′-GAATTGGGAATGCCCCAGTT-3′; ERVK pol F: 5′-TGATCCCMAAAGAYTGGCCTT-3′ and R: 5′-TTAAGCATTCCCTGAGGYAACA-3′; IRF1 F: 5′-AAAAGGAGCCAGATCCCAAGA-3′ and R: 5′-CATCCGGTACACTCGCACAG-3′. 18S rRNA was used as the endogenous control (Ambion kit #1718). The mean of two replicates per sample were analyzed using the ΔΔCT (Livak) method and normalized relative to calibrator sample control 03. GraphPad Prism was used to carry out statistical analyses including column statistics and unpaired t-test.

### Western Blots

PBMC were lysed on ice with 50 μl of in-house lysis buffer (0.05M Tris (pH 7.4), 0.15M NaCl, 0.002M EDTA, 10% glycerol and 1% NP-40 in ultra-pure water) to extract proteins. The lysis buffer was supplemented with 1x HALT protease and phosphatase inhibitor cocktail (Thermo Scientific #78442). BCA assay (Thermo Scientific #PI23227) was used to determine the protein content of each sample as per manufacturer's instructions. Cell lysates were prepared for SDS-PAGE and heated at 95°C for 10 min. Proteins (15 μg per lane) were separated by SDS-PAGE using a 10% BioRad Quick Cast gel (#161-0173) and transferred onto a PVDF membrane (BioRad #162-0260). The membrane was blocked in 5% skim milk solution for 30 min and probed with the desired primary antibody overnight at 4°C, followed by incubation at room temperature for 2 h. Primary antibodies used were: mouse anti-ERVK Gag (LifeSpan Biosciences # LS-C65287), rabbit anti-ERVK integrase (Pierce, custom antibody), rabbit anti-ERVK SU (Pierce, custom antibody), rabbit anti-human IRF1 (Santa Cruz #SC497), chicken anti-human β-actin (Abcam # ab13822; loading control). The membrane was probed with fluorophore-conjugated anti-mouse, chicken or anti-rabbit IgG secondary antibodies (1:1000 dilution; Molecular Probes #21449, A11072, A21246) for 2 h at room temperature. The membrane was imaged using a Protein Simple FluorChem M chemiluminescent imager, and multiplexed with readouts on the same blot in separate fluorescent channels. Image Lab software was used to determine the molecular weight and relative density (normalized to β-actin) of each band. The identity of each band was based on Gag-Pro-Pol processing, as previously described (24).

### Statistical Analyses

GraphPad Prism version 8.1.2 was used to carry out statistical analyses including column statistics and unpaired *t*-test used to assess clinical group differences for western blot and Q-PCR quantifications.

### Nomenclature

As with human genes, ERVK viruses (or HERV-K) are assigned names by the Human Gene Nomenclature Committee as recommended by Mayer et al. (25). Gene names in the text are italicized, whereas protein names are not.

## Results

### A Pilot Cohort of Patients With SBMA

Kennedy's disease is very rare and likely underestimated and underdiagnosed, with prevalence estimates of 1–2 per 100,000 individuals (26, 27). Founder effects are associated with regional increases in SBMA prevalence (28, 29).

With the goal of generating preliminary data for future studies, we recruited four patients with SBMA and four control participants to donate blood samples. The number of CAG repeats in the *AR* gene of patients with SBMA in this study is listed in Table 1. All SBMA cases had polyQ tracts extending beyond 38 CAG repeats, which is representative of pathological AR disruption (8).

### Control of ERVK Expression

**Figure 1:**
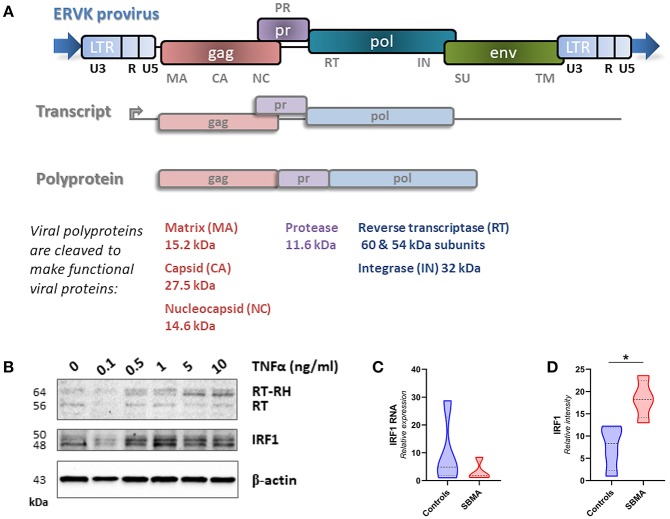
Genomic insertions of ERVK with intact open reading frames can produce a variety of mature viral proteins and are induced by pro-inflammatory signals. **(A)** The ERVK provirus is comprised of the typical retrovirus genes *gag, pro, pol*, and *env*. Multiple ERVK RNA transcripts are produced from a provirus. This diagram depicts the *gag*-*pro*-*pol* transcript. This transcript is translated to produce the Gag-Pro-Pol polyprotein, which is cleaved by the mature protease enzyme to produce individual viral proteins. Gag structural proteins (capsid, matrix and nucleocapsid) and *pol*-derived reverse transcriptase (RT/RT-RH heterodimer) integrase (IN) proteins were examined in this study. The cellular role of most ERVK proteins remains unknown; however, the ERVK Env protein is known to be neurotoxic (20). **(B)** ERVK reverse transcriptase (RT) levels dose-dependently increase with pro-inflammatory stimulus in immune cells. Peripheral blood mononuclear cells (PBMC) were treated with increasing doses of pro-inflammatory cytokine TNFα and evaluated for ERVK RT and IRF1 expression. As described elsewhere (19), the pro-inflammatory transcription factor IRF1 participates in driving increased levels of ERVK transcription and RT protein expression. **(C,D)** Similar IRF1 transcript expression in control and SBMA cases **(C)**, despite evidence of elevated IRF1 protein expression in SBMA as compared with controls (**D**, *p < 0.05).

The ERVK provirus is comprised of the typical retrovirus genes *gag, pro, pol*, and *env*. Multiple ERVK RNA transcripts are produced from a provirus. An essential transcript encoding both structural (Gag) and enzymatic (Pol) proteins is translated to produce the Gag-Pro-Pol polyprotein, which is cleaved by the mature protease enzyme to produce individual viral proteins (Figure 1). Protease cleavage sites in the ERVK Gag polyprotein are known, allowing the identification of mature viral proteins (30). Many inflammatory diseases are associated with elevated ERVK expression; although putative pathological contributions remain contentious (31, 32). Pro-inflammatory cytokine signaling has repeatedly been shown to contribute toward reactivation of endogenous retroviruses.

### IRF1 Expression Is Elevated in PBMC From Patients With SBMA

TNFα signaling can enhance IRF1 activity. The action of this inflammatory transcription factor has previously been implicated in the reactivation of ERVK (19). In PBMC, TNFα treatment dose-dependently increases both IRF1 and ERVK reverse transcriptase (RT) protein expression (Figure 1B). One notable observation in PBMC from patients with SBMA is a 2.4-fold enhanced expression of IRF1 as compared with controls (*p* < 0.05, Figure 1D), despite no evidence of differences in the IRF1 transcript between clinical groups (Figure 1C).

### ERVK Expression in PBMC From Patients With SBMA and Controls

**Figure 2:**
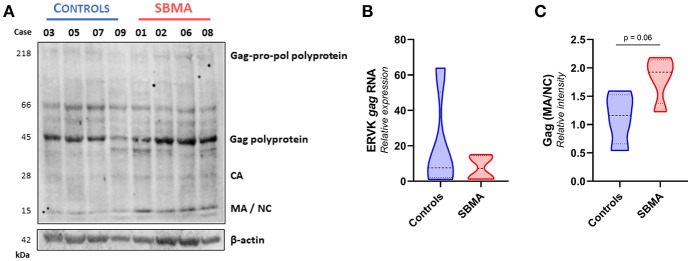
Patients with SBMA exhibit increased levels of mature ERVK Gag proteins. **(A)** *Ex vivo* PBMC from controls (*n* = 4) and patients with SBMA (*n* = 4) were evaluated for protein expression of ERVK Gag polyproteins and mature viral protein isoforms, capsid (CA), matrix (MA), and nucleocapsid (NC). Notable cleavage of ERVK polyproteins into mature protein forms occurs to a greater extent in patients with SBMA than controls. SBMA patients had 60, 58, 52, and 55 CAG repeats in their androgen receptor gene, respectively (left to right). Endogenous β-actin was used as a loading control. **(B)** Similar ERVK *gag* transcript expression in control and SBMA cases. **(C)** Relative quantification of Gag MA/NC bands revealed a trend toward increased processed structural viral protein expression between controls and patients with SBMA, with *p* = 0.06. Violin plots depict measured values from individual patients and the probability density of the grouped data.

**Figure 3:**
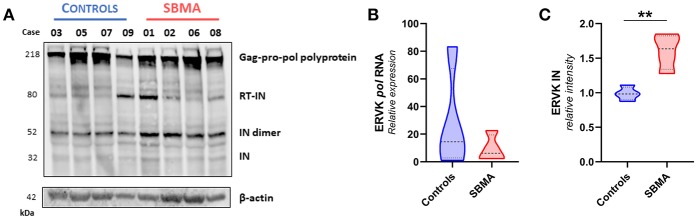
Patients with SBMA exhibit increased levels of ERVK integrase protein. **(A)** *Ex vivo* PBMC from controls (*n* = 4) and patients with SBMA (*n* = 4) were evaluated for protein expression of ERVK integrase (IN) polyproteins and mature isoform. Notable cleavage of ERVK pol polyprotein into mature 32 kDa monomer and 52 kDa dimer integrase forms occurs to a greater extent in patients with SBMA than controls. SBMA patients had 60, 58, 52, and 55 CAG repeats in their androgen receptor gene respectively (left to right). Endogenous β-actin was used as a loading control. **(B)** Similar ERVK *pol* transcript expression in control and SBMA cases. **(C)** Relative quantification of ERVK IN dimers (52 kDa bands) revealed statistical differences in IN protein expression between controls and patients with SBMA, with *p* < 0.01 (**). Violin plots depict measured values from individual patients and the probability density of the grouped data.

We assessed the protein expression of the Gag-Pro-Pol polyprotein (218 kDa), and its protease-derived cleavage products, by targeting either an N-terminal Gag epitope (Figure 2) or C-terminal integrase epitope (Figure 3) through a multiplex western blot analysis of *ex vivo* PBMC.

Basal expression of ERVK is expected in many human tissue types (33). Indeed, ERVK *gag* transcripts were readily measured in both controls and patients with SBMA, with no significant differences between clinical groups (Figure 2B). Figure 2A depicts that ERVK Gag polyproteins are evident in both controls and patients with SBMA. However, samples from patients with SBMA display more viral polyprotein processing leading to formation of mature viral proteins than their control counterparts. This is evident when examining the formation of mature structural Gag proteins, capsid (CA−28 kDa), matrix (MA−15 kDa) and nucleocapsid (NC−15 kDa). The sum of intensity quantification of MA/NC bands indicates that there is a trend toward more Gag polyprotein processing occurring in PBMC from patients with SBMA as compared with controls (Figure 2C, *p* = 0.06).

Figure 3 shows that there are similar levels of ERVK pol transcript (Figure 3B) and ERVK Gag-Pro-Pol polyprotein (218 kDa, Figure 3A) in PBMC from controls and SBMA. Similar to what was observed with the expression of ERVK Gag, the processing of viral proteins containing an integrase epitope was greater in SBMA samples than those of controls. Dimeric integrase is required for enzymatic activity (34); we observed monomeric (32 kDa) and an abundance of dimeric (52 kDa) integrase bands. Band intensity quantification of ERVK integrase protein indicates that there is significantly more Pol polyprotein processing occurring in PBMC from patients with SBMA as compared with controls (Figure 3C, *p* < 0.01).

Together these data show that ERVK viral protein maturation is enhanced in SBMA PBMC, as compared with control cells. The implications of ERVK viral protein activity as it may relate to SBMA will be discussed below.

## Discussion

PolyQ diseases highlight the complexity of translating the human genome into a given cellular state. The role of endogenous retroviruses further complicates matters. However, it is of critical importance to consider both cellular and viral contributors to disease processes. Here, for the first time we show evidence of ERVK viral protein maturation in SBMA. Albeit a preliminary study, our work points to additional avenues of investigation into the role of ERVK in this motor neuron disease.

### ERVK LTR and the AR Paradox

ERVK promoters contain binding sequences for AR. We have shown that 5′ and 3′ long terminal repeats (LTRs—viral promoters) which control ERVK expression contain conserved androgen response elements (35). Experimental evidence also supports a role for AR in enhancing ERVK expression (36). Therefore, we hypothesized that disrupted AR activity in SBMA would result in decreased ERVK protein levels; however, this was not the case. Our results consistently show that there is enhanced ERVK polyprotein processing and formation of mature (and potentially pathogenic) viral proteins in SBMA PBMC. This could be considered a novel gain-of-function in SBMA. Further validation using tissue samples from individuals with SBMA is warranted to confirm or refute a pathological impact from mature viral proteins, as ERVK expression in PBMC often co-occurs with evidence of viral proteins in other tissue types (37, 38).

### Inflammation as a Driver of ERVK Expression in SBMA

Inflammation and chronic immune stimulation are becoming recognized as a distinct feature of trinucleotide repeat expansion disorders (15). Peripheral immune activation is observed before clinical onset of Huntington's disease and in several murine models of polyQ disease (15, 39). Indeed, we observed enhanced expression of IRF1 in *ex vivo* (non-cultured/stimulated) PBMC from patients with SBMA, indicative of an ongoing inflammatory response. IRF1-dependent enhancement of inflammatory signaling is a potential underlying mechanism for ERVK expression in SBMA tissues, based on experimental cell culture models and observations in autopsied brain tissue specimens from patients with ALS (19).

### Failure to Degrade ERVK Proteins in SBMA?

Ubiquitination and digestion of unwanted cellular proteins—including protein aggregates and viral proteins—is crucial to maintain cellular homeostasis and is particularly important for neuronal health (40). Several studies indicate that similar to ALS, SBMA is also characterized by a failure in protein clearance mechanisms such as lysosomal degradation and autophagy (41, 42). Given that we observed similar levels of ERVK-derived transcripts between controls and patients with SBMA, the elevated ERVK polyprotein processing and mature ERVK proteins levels observed in SBMA may be related to a failure to degrade these viral proteins. Cell culture models show that inhibition of the proteasome can lead to an accumulation of ERVK proteins (16). ALS-associated mutations in TDP-43 are aggregate-prone and can facilitate the accumulation of ERVK proteins within cells (16). PolyQ expanded AR also forms proteinaceous toxic deposits in cells, which is associated with a loss-of-function as a transcriptional regulator (9, 43, 44). It remains unclear whether AR proteinopathy impacts the accumulation of ERVK proteins in SBMA.

### Potential Effects of ERVK on DNA Damage in SBMA

Several polyQ diseases exhibit evidence of heightened DNA damage and genomic instability (45, 46). In a murine model of SBMA, the extended polyQ tract in AR100 mice is associated with enhanced expression of DNA damage marker γH2AX in motor neurons, in conjunction with decreased expression of genes involved in DNA repair, such as *p53, Sesn1, ATR, Gadd45, Xrcc5*, and *Tp63* (42). Mutant AR protein can also act as a sink for DNA repair protein PTIP by sequestering it away from sites of DNA damage (47). Excessive ubiquitination of polyQ proteins may further compromise nuclear DNA repair processes through depletion of nuclear ubiquitin and histone de-ubiquitination (48). Enzymatic activity of retroviral integrase proteins can lead to significant DNA damage accumulation over time (49). Given a loss of DNA repair function in SBMA, this may render cells more vulnerable to DNA damaging insults like ERVK integrase activity (21) (unpublished data). Therefore, our observation of mature ERVK integrase protein expression in PBMC from patients with AR polyQ repeats is potentially pathologically relevant to the underlying multi-system disease processes that occur in SBMA.

## Conclusion

A novel gain-of-function effect in SBMA appears to be the enhancement of ERVK polyprotein processing into mature viral proteins in immune cells, despite an overall similar abundance of viral polyprotein in controls and patients with SBMA. While other ERVK-associated disease states exhibit increased levels of ERVK transcripts and viral protein (32), only viral protein processing seems to be altered in SBMA immune cells. As PBMC models do not necessarily reflect disease-relevant tissues, additional investigation into the role of endogenous retroviruses in SBMA is warranted. Should ERVK be further shown to contribute to the pathogenesis of SBMA, an antiviral therapeutic opportunity could be identified for this disease.

## Data Availability

All datasets [generated/analyzed] for this study are included in the manuscript and the supplementary files.

## Ethics Statement

Ethics approval was granted, and consent obtained from all participants in this study.

## Author Contributions

KS and RD conceived the project. KS obtained consent and collected the participant blood samples. CR, M-JN, and RD performed the experiments. RD wrote the manuscript.

### Conflict of Interest Statement

The authors declare that the research was conducted in the absence of any commercial or financial relationships that could be construed as a potential conflict of interest.

## Abbreviations

ALS: Amyotrophic Lateral Sclerosis
*AR*: Androgen receptor gene
AR: Androgen receptor protein
CA: Capsid
DNA: Deoxyribonucleic Acid
ERV: Endogenous Retrovirus
ERVK: Endogenous Retrovirus-K
HIV: Human Immunodeficiency Virus
IN: Integrase
LTR: Long Terminal Repeat
MA: Matrix
NC: Nucleocapsid
polyQ: polyglutamine tract caused by CAG nucleotide repeats
RNA: Ribonucleic Acid
SBMA: Spinal and bulbar muscular atrophy (Kennedy's disease)
SU: Surface Subunit of Envelope protein.

## References

[1] Atsuta N Watanabe H Ito M Banno H Suzuki K Katsuno M. Natural history of spinal and bulbar muscular atrophy (SBMA): a study of 223 Japanese patients. Brain. (2006) 129 (Pt 6), 1446–1455. 10.1093/brain/awl096 16621916

[2] Fischbeck KH. Spinal and bulbar muscular atrophy overview. J Mol Neurosci. (2016) 58:317–20. 10.1007/s12031-015-0674-7 26547319 PMC5094812

[3] Duyao M Ambrose C Myers R Novelletto A Persichetti F Frontali M. Trinucleotide repeat length instability and age of onset in Huntington's disease. Nat Genet. (1993) 4:387–92. 10.1038/ng0893-387 8401587

[4] Paulson HL Shakkottai VG Clark HB Orr HT. Polyglutamine spinocerebellar ataxias - from genes to potential treatments. Nat Rev Neurosci. (2017) 18:613–26. 10.1038/nrn.2017.92 28855740 PMC6420820

[5] Yu Z Zhu Y Chen-Plotkin AS Clay-Falcone D McCluskey L Elman L. PolyQ repeat expansions in ATXN2 associated with ALS are CAA interrupted repeats. PLoS ONE. (2011) 6:e17951. 10.1371/journal.pone.0017951 21479228 PMC3066214

[6] Chio A Calvo A Moglia C Canosa A Brunetti M Barberis M. ATXN2 polyQ intermediate repeats are a modifier of ALS survival. Neurology. (2015) 84:251–8. 10.1212/WNL.0000000000001159 25527265

[7] La Spada AR. Trinucleotide repeat instability: genetic features and molecular mechanisms. Brain Pathol. (1997) 7:943–63. 9217977 10.1111/j.1750-3639.1997.tb00895.x PMC8098141

[8] La Spada AR Wilson EM Lubahn DB Harding AE Fischbeck KH. Androgen receptor gene mutations in X-linked spinal and bulbar muscular atrophy. Nature. (1991) 352:77–9. 10.1038/352077a0 2062380

[9] Giorgetti E Lieberman AP. Polyglutamine androgen receptor-mediated neuromuscular disease. Cell Mol Life Sci. (2016) 73:3991–9. 10.1007/s00018-016-2275-1 27188284 PMC5045769

[10] Breza M Koutsis G. Kennedy's disease (spinal and bulbar muscular atrophy): a clinically oriented review of a rare disease. J Neurol. (2019) 266:565–73. 10.1007/s00415-018-8968-7 30006721

[11] Li M Miwa S Kobayashi Y Merry DE Yamamoto M Tanaka F. Nuclear inclusions of the androgen receptor protein in spinal and bulbar muscular atrophy. Ann Neurol. (1998) 44:249–54. 10.1002/ana.410440216 9708548

[12] Grunseich C Fischbeck KH. Spinal and bulbar muscular atrophy. Neurol Clin. (2015) 33:847–54. 10.1016/j.ncl.2015.07.002 26515625 PMC4628725

[13] Pennuto M Rinaldi C. From gene to therapy in spinal and bulbar muscular atrophy: are we there yet? Mol Cell Endocrinol. (2018) 465:113–21. 10.1016/j.mce.2017.07.005 28688959

[14] Huang S Zhu S Li XJ Li S. The expanding clinical universe of polyglutamine disease. Neuroscientist. (2019). 10.1177/1073858418822993. [Epub ahead of print]. 30614396 PMC6612477

[15] Olejniczak M Urbanek MO Krzyzosiak WJ. The role of the immune system in triplet repeat expansion diseases. Mediators Inflamm. (2015) 2015:873860. 10.1155/2015/873860 25873774 PMC4385693

[16] Manghera M Ferguson-Parry J Douville RN. TDP-43 regulates endogenous retrovirus-K viral protein accumulation. Neurobiol Dis. (2016) 94:226–36. 10.1016/j.nbd.2016.06.017. 27370226

[17] Bannert N Kurth R. Retroelements and the human genome: new perspectives on an old relation. Proc Natl Acad Sci USA. (2004) 101:14572–9. 10.1073/pnas.0404838101 15310846 PMC521986

[18] Cowley M Oakey RJ. Transposable elements re-wire and fine-tune the transcriptome. PLoS Genet. (2013) 9:e1003234. 10.1371/journal.pgen.1003234 23358118 PMC3554611

[19] Manghera M Ferguson-Parry J Lin R Douville RN. NF-kappaB and IRF1 induce endogenous retrovirus k expression via interferon-stimulated response elements in Its 5′ long terminal repeat. J Virol. (2016) 90:9338–49. 10.1128/JVI.01503-16 27512062 PMC5044829

[20] Li W Lee MH Henderson L Tyagi R Bachani M Steiner J. Human endogenous retrovirus-K contributes to motor neuron disease. Sci Transl Med. (2015). 7:307ra153. 10.1126/scitranslmed.aac8201 26424568 PMC6344353

[21] Bray S Turnbull M Hebert S Douville RN. Insight into the ERVK integrase - propensity for DNA damage. Front Microbiol. (2016) 7:1941. 10.3389/fmicb.2016.01941 27990140 PMC5131560

[22] Manghera M Ferguson-Parry J Douville RN. TDP-43 regulates endogenous retrovirus-K viral protein accumulation. Neurobiol Dis. (2016) 94:226–36. 10.1016/j.nbd.2016.06.017 27370226

[23] Douville RN Lissitsyn Y Hirschfeld AF Becker AB Kozyrskyj AL Liem J TLR4 Asp299Gly and Thr399Ile polymorphisms: no impact on human immune responsiveness to LPS or respiratory syncytial virus. PLoS ONE. (2010) 5:e12087 10.1371/journal.pone.0012087 20711470 PMC2919413

[24] Manghera M Ferguson J Douville R. ERVK polyprotein processing and reverse transcriptase expression in human cell line models of neurological disease. Viruses. (2015) 7:320–32. 10.3390/v7010320 25609305 PMC4306841

[25] Mayer J Blomberg J Seal RL. A revised nomenclature for transcribed human endogenous retroviral loci. Mob DNA. (2011) 2:7. 10.1186/1759-8753-2-7 21542922 PMC3113919

[26] Mariotti C Castellotti B Pareyson D Testa D Eoli M Antozzi C. Phenotypic manifestations associated with CAG-repeat expansion in the androgen receptor gene in male patients and heterozygous females: a clinical and molecular study of 30 families. Neuromuscul Disord. (2000) 10:391–7. 10.1016/S0960-8966(99)00132-7 10899444

[27] Guidetti D Sabadini R Ferlini A Torrente I. Epidemiological survey of X-linked bulbar and spinal muscular atrophy, or Kennedy disease, in the province of Reggio Emilia, Italy. Eur J Epidemiol. (2001) 17:587–91. 10.1023/A:1014580219761 11949733

[28] Tanaka F Doyu M Ito Y Matsumoto M Mitsuma T Abe K. Founder effect in spinal and bulbar muscular atrophy (SBMA). Hum Mol Genet. (1996) 5:1253–7. 10.1093/hmg/5.9.1253 8872464

[29] Udd B Juvonen V Hakamies L Nieminen A Wallgren-Pettersson C Cederquist K. High prevalence of Kennedy's disease in Western Finland – is the syndrome underdiagnosed? Acta Neurol Scand. (1998) 98:128–33. 9724012 10.1111/j.1600-0404.1998.tb01732.x

[30] Kraus B Boller K Reuter A Schnierle BS. Characterization of the human endogenous retrovirus K Gag protein: identification of protease cleavage sites. Retrovirology. (2011) 8:21. 10.1186/1742-4690-8-21 21429186 PMC3073897

[31] Bannert N Hofmann H Block A Hohn O. HERVs new role in cancer: from accused perpetrators to cheerful protectors. Front Microbiol. (2018) 9:178. 10.3389/fmicb.2018.00178 29487579 PMC5816757

[32] Garcia-Montojo M Doucet-O'Hare T Henderson L Nath A. Human endogenous retrovirus-K (HML-2): a comprehensive review. Crit Rev Microbiol. (2018) 44:715–38. 10.1080/1040841X.2018.1501345 30318978 PMC6342650

[33] Seifarth W Frank O Zeilfelder U Spiess B Greenwood AD Hehlmann R. Comprehensive analysis of human endogenous retrovirus transcriptional activity in human tissues with a retrovirus-specific microarray. J Virol. (2005) 79:341–52. 10.1128/JVI.79.1.341-352.2005 15596828 PMC538696

[34] Delelis O Parissi V Leh H Mbemba G Petit C Sonigo P. Efficient and specific internal cleavage of a retroviral palindromic DNA sequence by tetrameric HIV-1 integrase. PLoS ONE. (2007) 2:e608. 10.1371/journal.pone.0000608 17622353 PMC1905944

[35] Manghera M Douville RN. Endogenous retrovirus-K promoter: a landing strip for inflammatory transcription factors? Retrovirology. (2013) 10:16. 10.1186/1742-4690-10-16 23394165 PMC3598470

[36] Hanke K Chudak C Kurth R Bannert N. The rec protein of HERV-K(HML-2) upregulates androgen receptor activity by binding to the human small glutamine-rich tetratricopeptide repeat protein (hSGT). Int J Cancer. (2013) 132:556–67. 10.1002/ijc.27693 22733359

[37] Wallace TA Downey RF Seufert CJ Schetter A Dorsey TH Johnson CA. Elevated HERV-K mRNA expression in PBMC is associated with a prostate cancer diagnosis particularly in older men and smokers. Carcinogenesis. (2014) 35:2074–83. 10.1093/carcin/bgu114 24858205 PMC4146419

[38] Douville RN Nath A. Human endogenous retrovirus-K and TDP-43 expression bridges ALS and HIV neuropathology. Front Microbiol. (2017) 8:1986. 10.3389/fmicb.2017.01986 29075249 PMC5641584

[39] Bjorkqvist M Wild EJ Thiele J Silvestroni A Andre R Lahiri N. A novel pathogenic pathway of immune activation detectable before clinical onset in Huntington's disease. J Exp Med. (2008) 205:1869–77. 10.1084/jem.20080178 18625748 PMC2525598

[40] Hallengren J Chen PC Wilson SM. Neuronal ubiquitin homeostasis. Cell Biochem Biophys. (2013) 67:67–73. 10.1007/s12013-013-9634-4 23686613 PMC3758786

[41] Rusmini P Crippa V Cristofani R Rinaldi C Cicardi ME Galbiati M. The role of the protein quality control system in SBMA. J Mol Neurosci. (2016) 58:348–64. 10.1007/s12031-015-0675-6 26572535

[42] Malik B Devine H Patani R La Spada AR Hanna MG Greensmith L. Gene expression analysis reveals early dysregulation of disease pathways and links Chmp7 to pathogenesis of spinal and bulbar muscular atrophy. Sci Rep. (2019) 9:3539. 10.1038/s41598-019-40118-3 30837566 PMC6401132

[43] Orr CR Montie HL Liu Y Bolzoni E Jenkins SC Wilson EM. An interdomain interaction of the androgen receptor is required for its aggregation and toxicity in spinal and bulbar muscular atrophy. J Biol Chem. (2010) 285:35567–77. 10.1074/jbc.M110.146845 20826791 PMC2975181

[44] Berger TR Montie HL Jain P Legleiter J Merry DE. Identification of novel polyglutamine-expanded aggregation species in spinal and bulbar muscular atrophy. Brain Res. (2015) 1628 (Pt B), 254–264. 10.1016/j.brainres.2015.09.033 26453288 PMC4681586

[45] Giuliano P De Cristofaro T Affaitati A Pizzulo GM Feliciello A Criscuolo C. DNA damage induced by polyglutamine-expanded proteins. Hum Mol Genet. (2003) 12:2301–9. 10.1093/hmg/ddg242 12915485

[46] Illuzzi J Yerkes S Parekh-Olmedo H Kmiec EB. DNA breakage and induction of DNA damage response proteins precede the appearance of visible mutant huntingtin aggregates. J Neurosci Res. (2009) 87:733–47. 10.1002/jnr.21881 18831068

[47] Xiao H Yu Z Wu Y Nan J Merry DE Sekiguchi JM. A polyglutamine expansion disease protein sequesters PTIP to attenuate DNA repair and increase genomic instability. Hum Mol Genet. (2012) 21:4225–36. 10.1093/hmg/dds246 22736030 PMC3441122

[48] Ben Yehuda A Risheq M Novoplansky O Bersuker K Kopito RR Goldberg M. Ubiquitin accumulation on disease associated protein aggregates is correlated with nuclear ubiquitin depletion, histone de-ubiquitination and impaired DNA damage response. PLoS ONE. (2017) 12:e0169054. 10.1371/journal.pone.0169054 28052107 PMC5215683

[49] Daniel R Ramcharan J Rogakou E Taganov KD Greger JG Bonner W. Histone H2AX is phosphorylated at sites of retroviral DNA integration but is dispensable for postintegration repair. J Biol Chem. (2004) 279:45810–4. 10.1074/jbc.M407886200 15308627

